# Intra- and Trans-Generational Costs of Reduced Female Body Size Caused by Food Limitation Early in Life in Mites

**DOI:** 10.1371/journal.pone.0079089

**Published:** 2013-11-12

**Authors:** Andreas Walzer, Peter Schausberger

**Affiliations:** Group of Arthropod Ecology and Behavior, Division of Plant Protection, Department of Crop Sciences, University of Natural Resources and Life Sciences, Vienna, Austria; Université Paris 13, France

## Abstract

**Background:**

Food limitation early in life may be compensated for by developmental plasticity resulting in accelerated development enhancing survival at the expense of small adult body size. However and especially for females in non-matching maternal and offspring environments, being smaller than the standard may incur considerable intra- and trans-generational costs.

**Methodology/Principal Findings:**

Here, we evaluated the costs of small female body size induced by food limitation early in life in the sexually size-dimorphic predatory mite *Phytoseiulus persimilis*. Females are larger than males. These predators are adapted to exploit ephemeral spider mite prey patches. The intra- and trans-generational effects of small maternal body size manifested in lower maternal survival probabilities, decreased attractiveness for males, and a reduced number and size of eggs compared to standard-sized females. The trans-generational effects of small maternal body size were sex-specific with small mothers producing small daughters but standard-sized sons.

**Conclusions/Significance:**

Small female body size apparently intensified the well-known costs of sexual activity because mortality of small but not standard-sized females mainly occurred shortly after mating. The disadvantages of small females in mating and egg production may be generally explained by size-associated morphological and physiological constraints. Additionally, size-assortative mate preferences of standard-sized mates may have rendered small females disproportionally unattractive mating partners. We argue that the sex-specific trans-generational effects were due to sexual size dimorphism – females are the larger sex and thus more strongly affected by maternal stress than the smaller males – and to sexually selected lower plasticity of male body size.

## Introduction

Potential immediate negative implications of food limitation during the juvenile phase are often mitigated by developmental plasticity with profound effects on fitness-relevant, commonly interrelated morphological, physiological and behavioral traits at maturity [1], [2]. The mode of food stress determines which of the two pivotal life history traits - age and size at maturity - should be traded off against the other. When juveniles are exposed to a limited infinite, i.e. constant food resource, prolonged development may allow reaching standard body size at the expense of later maturity [1]. When early developmental stress arises from a limited finite, i.e. diminishing food resource, selection should favour accelerated development enhancing juvenile survival at the expense of smaller body size at maturity [3]. Such a developmental plasticity pattern is typical for species that are well adapted to ephemeral food resources such as the desert amphibian *Scaphiopus couchii* [4], the dung fly *Scathophaga stercoraria* [5], the seed beetle *Callosobruchus maculatus* [6] and the predatory mites *Phytoseiulus persimilis* and *Neoseiulus californicus* [7].

However and particularly in sexually size-dimorphic species, developmental plasticity leading to smaller than standard-sized adult females incurs also costs [8] because body size affects numerous fitness-relevant traits of adult females. First, female longevity is an important factor influencing reproductive success [9], [10], and female body size is often positively correlated to longevity [11], [12]. For example, small females of the butterfly *Pieris rapae* had a lower reproductive success relative to standard-sized females because of shorter longevities [13]. Second, small females may be less often chosen by males for mating than standard-sized females if males use female body size as indicator of mate quality [14]. Empirical evidence comes from vertebrates (amphibians [15], [16]) and invertebrates (insects [17], [18]). Third, small females often produce smaller eggs and/or a lower number of eggs than standard-sized females, which is well documented for arthropods such as orb-weaving spiders [19], dipterans [20], [21], lepidopterans [22], [23], coleopterans [24] and orthopterans [25].

Small female body size induced by food limitation during juvenile development does not only change within-generation performance but commonly exerts also trans-generational effects on the offspring. Trans-generational or maternal effects are defined as any effect on the offspring phenotype by environmental experience of their mothers, which is independent from the direct effects of the transmitted genes [26], [27]. Early maternal developmental stress may for example influence offspring body size [28], [29], developmental time [30], [31], fecundity and survival [32]. Additionally, maternal effects may be apparent in one, but not in the other offspring sex [28], [29]. Sex-specific maternal effects should be more likely in species with sexual egg size dimorphism, which is documented for birds, reptiles and mites (reviewed in [33]). Implying that small females produce smaller eggs compared to standard-sized females, then the offspring sex arising from larger eggs should have larger disadvantages relative to the offspring sex arising from smaller eggs [1].

Here, we studied the intra- and trans-generational effects of female body size on fitness-relevant maternal and offspring traits in the plant-inhabiting predatory mite *Phytoseiulus persimilis*, which is a specialized predator of spider mites of the genus *Tetranychus* [34]. Spider mites constitute an ephemeral and patchily distributed food resource for *P. persimilis* with rapidly succeeding phases of host plant colonisation, population increase, dispersal and local extinction [35]. Consequently, juvenile *P. persimilis* are often confronted with food limitation because they tend to stay in limited finite spider mite patches until adulthood. However, food limitation affects the age and size at maturity of the developmentally plastic *P. persimilis*: under limited food *P. persimilis* females reach earlier adulthood increasing their survival probabilities at the expense of smaller body size [7]. *Phytoseiulus persimilis* is sexually dimorphic with smaller males emerging from smaller eggs [7], [36]. The tertiary sex ratio (proportion of females at sexual maturity) is under maternal control and female-biased ranging from 0.6 to 0.8 in dependence of the environmental conditions [37]. A single mating per lifetime is sufficient for maximum egg production [38]. Maternal care is restricted to oviposition site selection based primarily on prey availability and offspring predation risk [39]–[41]. The reproductive period may last several weeks and daily egg production mainly depends on prey supply of the adult females [42], [43], making *P. persimilis* a typical income breeder [44]. Thus, the longevity of a reproductive female is a decisive determinant of her reproductive success. In detail, we evaluated the costs of small female body size induced by food limitation early in life on their longevity, attractiveness as mates, number and size of eggs, offspring sex ratio, offspring survival and sex-specific offspring body size. We pursued three major hypotheses: (1) small females are less vigorous and are thus less attractive for male mating partners; (2) small maternal body size reduces the number and/or size of eggs; (3) the effects of small maternal body size are projected into the next generation affecting the large-sized daughters more strongly than the small-sized sons.

## Materials and Methods

### Predator Rearing and Experimental Units

Specimens of *P. persimilis* used to found a laboratory-reared population were collected from various herbs in a non-agricultural, non-protected area in the southwest of Palermo (State Trapani, Sicily) in 2007 [41], [45]. *P. persimilis* is not a protected or endangered species. The predators were reared on a plastic tile resting on a water-saturated foam cube (∼15×15 cm) in a plastic box half-filled with water [7] and fed with two-spotted spider mites, *Tetranychus urticae,* by adding spider mite-infested bean leaves onto arenas in two to three day intervals.

We used two types of experimental units. (1) Closable acrylic cages, consisting of cylindrical cells of 15 mm diameter and 3 mm height with a fine mesh screen at the bottom and closed on the upper side with a microscope slide fixed by a rubber band [46], were used for generating small and standard-sized predator females, and for the mating behaviour experiments. (2) Detached bean leaf arenas were used to evaluate the influence of female body size on survival, egg number and size, offspring survival, sex ratio and body size. Each leaf arena consisted of a single detached bean leaf (∼4 cm^2^) placed upside down on a water-saturated foam cube (∼5×5 cm) in a plastic box half-filled with water. The leaf arena was delimited by strips of moist tissue paper preventing the mites from escaping.

### Induction of Female Body Size Plasticity

To obtain small and standard-sized females used in experiments, eggs of *P. persimilis* were randomly taken from the rearing unit and singly reared in closed acrylic cages with either limited (10 spider mite eggs) or ample (>50 spider mite eggs) prey [7]. The developmental progress of the juvenile predators was checked every 24 h. Irrespective of food supply, all juvenile predators reached adulthood excluding potential effects of body size specific mortality. Then the sex of the mites was visually determined (males are approximately 20–30% smaller than females). The virgin females generated by limited and ample prey supply were used in the experiments as small and standard-sized females, respectively. To validate the body size differences, the females were mounted in a drop of Hoyer’s medium [47] after termination of the experiments. After drying the microscope slides at room conditions for two days, the dorsal shield lengths of the females were measured under the microscope at 200x magnification. Dorsal shield length is a suitable indicator of body size in phytoseiid mites [48].

### Mate Choice

A small and a standard-sized virgin female were placed together with a male, randomly chosen from the rearing unit, in an acrylic cage and provided with ample spider mite prey (eggs and juveniles). To distinguish between small and standard-sized females, they were marked with red and blue water-color dots on their dorsal shields before the experiment. Colors were randomly assigned to small and standard-sized females. After loading, the cages were checked every 10 to 15 min for 3 h to determine which female (small or standard-sized) mated first. 18 replicates, i.e. pairs of small and standard-sized females, were conducted.

### Female Survival, Mating Behavior and Fecundity

Small or standard-sized females were singly placed into acrylic cages together with a male randomly taken from the rearing unit and provided with ample spider mite prey. The mating behavior of each couple was monitored every 10 to 15 min until the first mating was finished (mean mating duration is 120 to 180 min [38]). Subsequently, the mated females were singly placed on detached bean leaves infested with ample spider mite prey. The state of the females (dead, alive) was checked and the deposited eggs were counted and removed every 24 h over 10 consecutive days. Spider mite prey was replenished if necessary by brushing spider mites from infested leaves onto the arena. 14 replicates per female size category were conducted. Additionally, width (a) and length (b) of the eggs produced by the females were measured immediately after collection under a microscope with 200x magnification without destroying the eggs. The average egg volume (V) per female per day was calculated by the formula for a prolate spheroid:
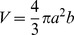



### Offspring Survival to Adulthood, Sex-ratio and Body Size

All eggs of each mated small and standard-sized female were placed on separate detached bean leaf arenas with ample spider mite prey and left to develop. After reaching adulthood all offspring of each female were sexed, mounted in a drop of Hoyer’s medium and their dorsal shield length measured under the microscope at 200x magnification. The sex-specific average dorsal shield length of the offspring was calculated for each small and standard-sized mother.

### Statistical Analyses

SPSS 18.0.1 (SPSS Inc., 2006) was used for all statistical analyses. All data are available as supplementary material (Data File S1). The proportion of males mated with a small or standard-sized female in the choice experiment was compared by a two-sided binomial test assuming equal choice. The survival functions of small and standard-sized females (combination of cumulative survival and survival time) provided with ample spider mite prey over 10 days were compared by Breslow tests within the Kaplan-Meier procedure [49]. Generalized linear models (GLM) were used to analyse the effects of female body size (small, standard-sized) on the time of first mating, the mating duration, the pre-oviposition period, offspring survival and offspring sex ratio (normal distribution, identity link function). Proportional offspring survival and sex ratio data were arcsin-square root transformed before analysis. Similarly, the influence of maternal body size and offspring sex on offspring body size was analysed by GLM (normal distribution, identity link function). Generalized estimating equations (GEE, normal distribution with identity link function, autocorrelation structure between observation days) were used to compare the influence of maternal body size and time (used as within subject variable) on egg number (covariate: egg volume) and volume (covariate: egg number). To detail changes over time within and between maternal body size categories, pairs of the estimated marginal means were compared by least significant difference (LSD) tests. T-tests for dependent samples were used to compare the maternal and daughterly body sizes within each maternal size category (small and standard-sized).

## Results

### Induction of Female Body Size Plasticity

The mean dorsal shield length of females provided with limited prey was significantly smaller than that of females provided with ample prey (mate choice experiment, T-test for independent samples: *T*_34_ = −8.641, *p*<0.001; mating behavior experiment: *T*_26_ = −7.452, *p*<0.001) (Table 1).

**Table 1:** Intra- and trans-generational effects of maternal food conditions during early life on maternal and offspring body size (dorsal shield length in µm, mean ± SE).

Sex/generation	Food-limited^1^	Non food-limited^1^
Mother	317.43±2.62^aA^	340.55±0.94^bA^
Son^2^	275.28±0.67^a^	275.31±0.49^a^
Daughter^2^	329.72±0.89^aB^	339.36±1.64^bA^

1 Different small superscript letters within rows indicate significant differences between food conditions for a given sex/generation (GLM: *p*<0.05); different capital superscript letters within columns indicate significant differences between mothers and daughters within a given food condition (T-tests for dependent samples: *p*<0.05).

2 Offspring were always provided with ample prey but derived from food- or non food-limited mothers.

### Mate Choice

Small female body size significantly decreased female attractiveness to males and/or the likelihood of being chosen by a male (two-sided binomial test: *p* = 0.001). In choice situations, 16 out of 18 males mated first with the standard-sized female.

### Female Survival, Mating Behavior and Fecundity

**Figure 1:**
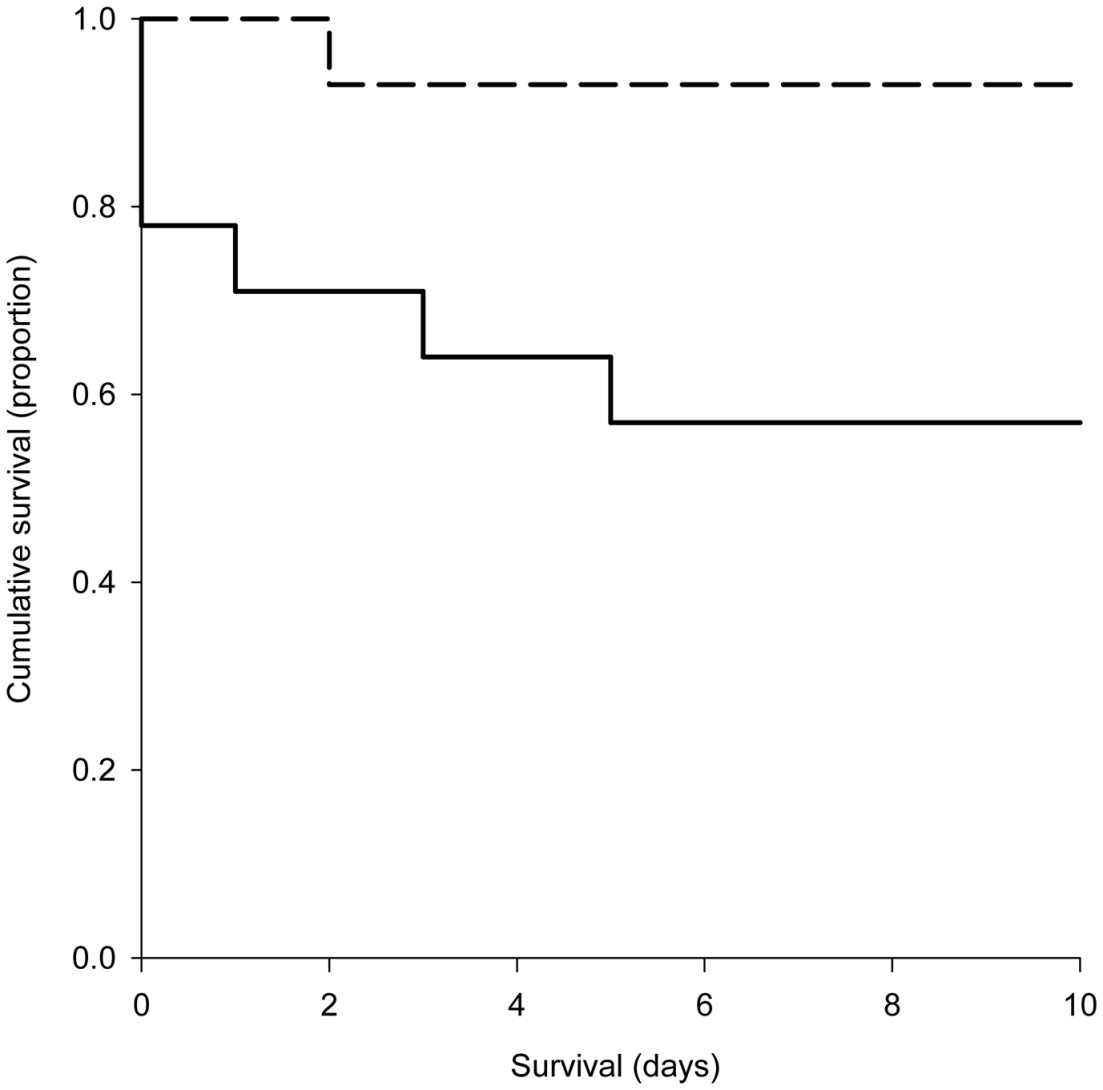
Body size effects on female survival. The survival function (combination of cumulative survival and survival time) of small *P. persimilis* females (solid line) was lower than that of standard-sized females (dashed line) when provided with ample spider mite prey over 10 days.

Small body size lowered the survival functions of adult females (combination of cumulative survival and survival time) (*χ^2^*_1_ = 4.776, *p* = 0.029). Only 1 out of 14 standard-sized females but 6 out of 14 small females died during the experimental 10 days oviposition period. Moreover, 3 small adult females died after mating before beginning oviposition (Figure 1).

**Figure 2:**
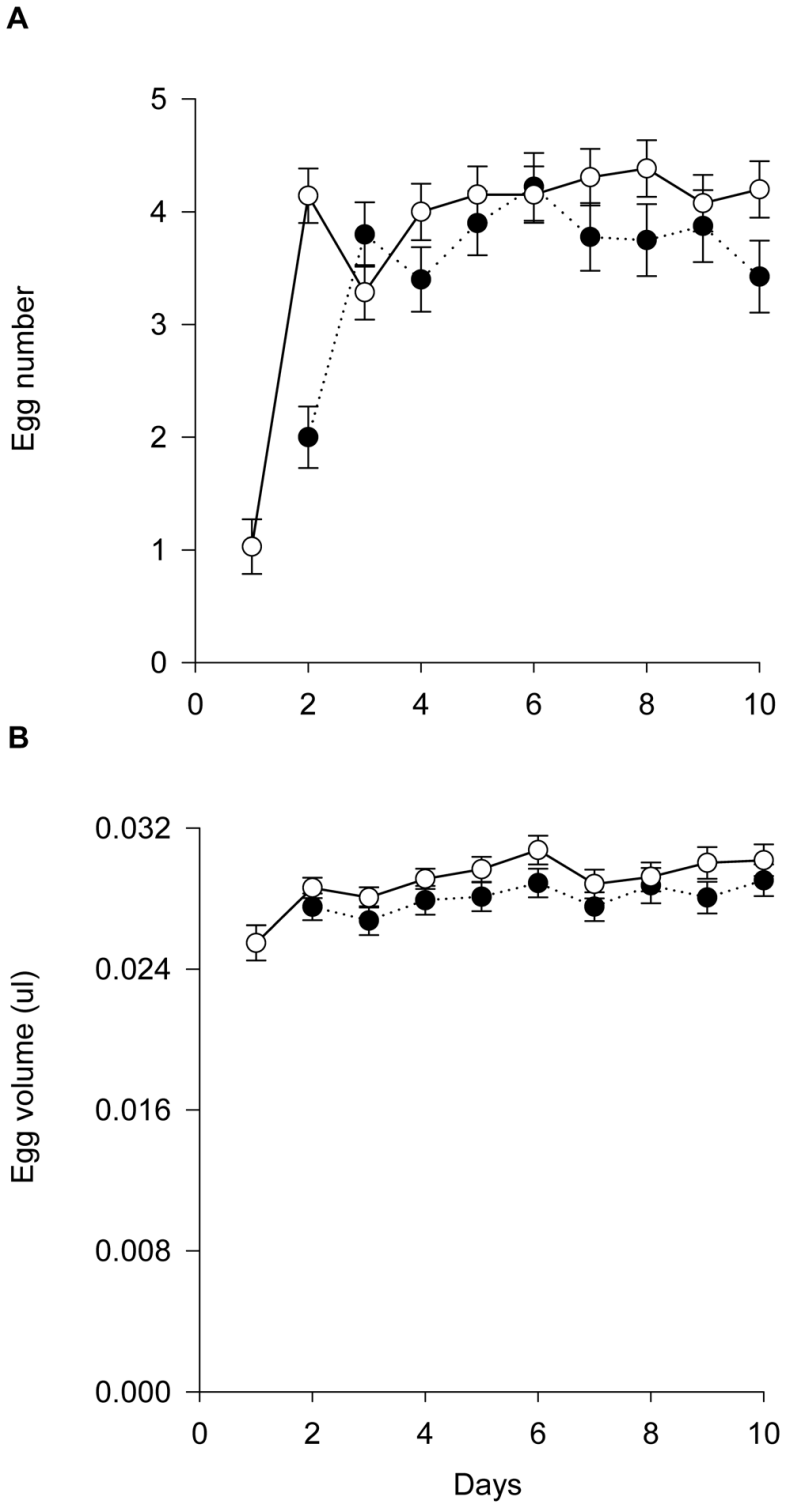
Maternal body size effects on egg number and size. Small *P. persimilis* females (dotted line, open circles) produced fewer (A) and smaller (B) eggs (mean ± SE) than standard-sized females (solid line, closed circles) when provided with ample spider mite prey over 10 days.

Female body size did not affect the latency to first mating (mean ± SE; small females: 33.43±4.88 min, standard-sized females: 29.93±5.59; GLM: Wald *χ^2^*_1_ = 0.240, *p* = 0.625) and the mating duration (mean ± SE; small females: 168.00±8.97 min, standard-sized females: 169.93±13.15; Wald *χ^2^*_1_ = 0.016, *p* = 0.900). However, small female body size elongated the pre-oviposition period, i.e. the time elapsed between mating and laying the first egg, (Wald *χ^2^*_1_ = 52.762, *p*<0.001; mean ± SE; small females: 1.59±0.10 days; standard-sized females: 0.64±0.09). The number of eggs produced per day was not affected by egg volume (GEE: Wald *χ^2^*_1_ = 0.098, *p* = 0.754) but by female body size (Wald *χ^2^*_1_ = 28.508, *p*<0.001) and time (Wald *χ^2^*_9_ = 862.813, *p*<0.001). Moreover, egg production progressed differently over time, indicated by the interaction between egg number and time (Wald *χ^2^*_9_ = 43.162, *p*<0.001). Small females produced fewer eggs and reached the oviposition plateau about one day later than standard-sized females (Figure 2A). Also the egg volume was not affected by the number of eggs (GEE: Wald *χ^2^*_1_ = 0.974, *p* = 0.324) but by maternal body size (GEE: Wald *χ^2^*_1_ = 19.946, *p*<0.001) and time (Wald *χ^2^*_9_ = 111.036, *p*<0.001). The interaction of time and female body size did not influence the egg volume (Wald *χ^2^*_8_ = 6.107, *p* = 0.635). Across time, small females produced less voluminous eggs than standard-sized females. Pooled over both female body size categories, eggs produced on the first day were less voluminous than those produced later (pairwise post hoc comparisons, LSD: *p*<0.05) (Figure 2B).

### Offspring Survival to Adulthood, Sex-ratio and Body Size

Maternal body size did not influence offspring sex ratio (proportion of females, mean ± SE; small females: 0.75±0.05; standard-sized females: 0.71±0.05; GLM: Wald *χ^2^*_1_ = 0.591, *p* = 0.442) and offspring proportional survival to adulthood (mean ± SE; small females: 0.93±0.03, standard-sized females: 0.94±0.04; GLM: Wald *χ^2^*_1_ = 0.321, *p* = 0.571). Maternal body size (GLM: Wald *χ^2^*_1_ = 16.902, *p*<0.001) and offspring sex (Wald *χ^2^*_1_ = 5197.421, *p*<0.001) determined offspring body size. The effect of maternal body size was dependent on offspring sex (body size by sex interaction: Wald *χ^2^*_1_ = 16.561, *p*<0.001). Female but not male offspring body size was affected by maternal body size, with small mothers producing smaller daughters than standard-sized mothers (Table 1). Daughters from small mothers were larger than their mothers (T-test for dependent samples: *T*_10_ = 5.638, *p*<0.001), whereas standard-sized mothers and their daughters were similarly sized (*T*_13_ = 0.640, *p* = 0.533) (Table 1).

## Discussion

Our experiments revealed both intra- and trans-generational costs of reduced female body size, caused by food limitation early in life, in the predatory mite *P. persimilis*. Small body size negatively influenced the survival probabilities of the adult females and severely reduced their attractiveness to potential mating partners. Both egg size and number were positively correlated with maternal body size. The effect of maternal body size on offspring body size was sex-specific. Daughters but not sons of small mothers were smaller than those from standard-sized mothers.

### Intra-generational Effects of Small Female Body Size

Female body size strongly influenced the outcome of the male mate choice experiment with almost all males preferentially mating with standard-sized females. This finding does not necessarily mean that small females never obtain a mating partner under natural conditions. The primary sex-ratio of *P. persimilis* is strongly female-biased. However, a single mating suffices for maximum egg production [38], whereas males are highly polygynous with each male fertilizing ∼50 females [50]. Consequently, the operational sex ratio (the number of receptive females per sexually active male) is extremely low increasing the chances of small *P. persimilis* females to obtain a mate.

Several proximate reasons may be put forward to explain the inferiority of small females in the male mate choice experiments. Non-random mating can be the result of assortative or disassortative matching of male and female phenotypes. Males used in the choice experiments likely were standard-sized because they are only little plastic in body size at maturity [7] and were randomly withdrawn from the rearing unit, where they were provided with ample food. Accordingly, size-assortative mating could have taken place with a directional preference of standard-sized males or females for standard-sized mates [51]. However, there is no size-assortative mating in *P. persimilis* (Walzer and Schausberger, unpublished). Likely, small body size induced by early food stress is associated with physiological constraints. For example, small body size may have resulted in less or lower quality sex pheromones released by the females. Overall, the females could have been less vigorous than standard-sized females. There is considerable evidence that sexual activity shortens the longevity of females in several arthropod species including mites [52]–[54]. Small female body size may have amplified these costs in our experiments because some small but not standard-sized *P. persimilis* females died soon after mating without producing a single egg. The other small females started with the production of the first eggs not before the third day, whereas standard-sized females produced their first egg much earlier. The number of produced eggs during a female’s lifetime is positively correlated with the mating duration in *P. persimilis* [50], which was not affected by female body size. Hence, it is likely that the females were provided with similar quantities of sperm material independent of their body size. However, in addition to producing fewer eggs, small females produced also smaller eggs than standard-sized females. Such a pattern is known from many arthropod taxa such as mosquitoes, wasps, bugs, moths, butterflies and beetles (reviewed in [11]). Ultimately, small females may have tried to keep up the number of eggs at the expense of egg size. Alternatively, small egg size may have simply been caused by morphological and physiological constraints of small females relative to standard-sized females [11].

### Sex-specific Effects of Maternal Body Size on Offspring Body Size

Maternal body size was only correlated with female but not male offspring body size: small mothers produced small daughters but standard-sized sons. Similarly, early maternal developmental stress in the zebra finch *Taeniopygia guttata* caused small body size in daughters but not sons [29]. The mechanisms resulting in small daughters of *P. persimilis* and *T. guttata*, however, seem to be species-specific based on differing biological and ecological traits. Parental care by nestling feeding is common in size monomorphic zebra finches [55], [56], and small mothers probably reduce parental care [29]. Nutritional stress affects daughters more strongly than sons in zebra finches, because of their higher metabolic costs [57], which may have resulted in sex-specific trans-generational effects on offspring body size in *T. guttata*. In contrast, there is no maternal food provisioning after egg deposition in *P. persimilis*. In *P. persimilis*, it may simply go with the commonly observed trend that the larger sex is more strongly affected by maternal stress than the smaller sex [1]. Alternatively or additionally, the mating and reproductive costs of deviations from standard body size are more severe in males than females leading to stronger canalization of male body size [58].

### Benefits and Costs of Sex-specific Trans-generational Body Size Effects

When the offspring environment matches the environment experienced by their mothers, trans-generational effects are expected to have a net benefit on offspring fitness [26]. Small females need less food for maintenance of basic metabolism and reproduction than standard-sized females. However, there are also examples reporting adverse effects of early maternal food stress on offspring fitness under matching environmental conditions [31], [59]. Examples of trans-generational effects caused by early maternal food stress provide evidence that the size of eggs produced by food stressed small mothers relative to the eggs produced by unstressed standard-sized mothers is decisive for a net fitness benefit to occur or not. Small *Drosophila melanogaster* females were able to produce large eggs and their offspring developed faster at the expense of slightly smaller body size under resource-poor environmental conditions [60]. Under resource-rich environmental conditions the offspring were even larger than offspring derived from standard-sized females [30]. Contrary, in the neriid fly *Telostylinus angusticollis,* small females produced smaller eggs than standard-sized females. Offspring body size was not affected by egg size under resource-poor environmental conditions but offspring arising from small eggs had longer developmental times [31]. In our experiments, small *P. persimilis* females produced small eggs and their daughters were also small although they were reared under resource-rich environmental conditions. Statistical analysis revealed that daughters from small mothers were smaller than daughters from standard-sized mothers but they were larger than their mothers. Therefore, being born in and growing up under favorable environmental conditions seems to alleviate the costs associated with trans-generational carry-over effects of small maternal body size. Presumably, but this needs to be tested, the trans-generational effects of small maternal body size could completely disappear in the second or third filial generation under persisting environmental resource-richness.

## Supporting Information

Data File S1 **Data from the experiments presented in separate working sheets.** Working sheet (WS) 1: female body size plasticity; WS 2: mate choice; WS 3: female survival, mating; WS 4: egg number, volume; WS 5: offspring size, sex, survival; WS 6: mother daughter size.
(XLSX)

## References

[1] Nylin S, Gotthard K (1998) Plasticity in life-history traits. Annu Rev Entomol 43: 63–83. 9444750 10.1146/annurev.ento.43.1.63

[2] Snell-Rood EC (2013) An overview of the evolutionary causes and consequences of behavioural plasticity. Anim Behav 85: 1004–1011.

[3] Abrams PA, Leimar O, Nylin S, Wiklund C (1996) The effect of flexible growth rates on optimal sizes and development times in a seasonal environment. Am Nat 147: 381–395.

[4] Newman RA (1994) Effects of changing density and food level on metamorphosis of a desert amphibian, *Scaphiopus couchii* . Ecology 75: 1085–1096.

[5] Blanckenhorn WU (1999) Different growth responses to temperature and resource limitation in three fly species with similar life histories. Evol Ecol 13: 395–409.

[6] Møller H, Smith RH, Sibly RM (1990) Evolutionary demography of a bruchid beetle. III. Correlated responses to selection and phenotypic plasticity. Funct Ecol 4: 489–493.

[7] Walzer A, Schausberger P (2011) Sex-specific developmental plasticity of generalist and specialist predatory mites (Acari: Phytoseiidae) in response to food stress. Biol J Linn Soc 102: 650–660. 10.1111/j.1095-8312.2010.01593.x PMC3191859 22003259

[8] Metcalfe NB, Monaghan P (2001) Compensation for a bad start: grow now, pay later? Trends Ecol Evol 16: 254–260. 11301155 10.1016/s0169-5347(01)02124-3

[9] Robbins AM, Stoinski T, Fawcett K, Robbins MM (2011) Lifetime reproductive success of female mountain gorillas. Am J Phys Anthrop 146: 582–593. 21989942 10.1002/ajpa.21605

[10] Herenyi M, Hegyi G, Garmszegi LZ, Hargitai R, Michl G, et al (2012) Lifetime offspring production in relation to breeding lifespan, attractiveness, and mating status in male collared flycatchers. Oecologia 170: 935–942. 22644049 10.1007/s00442-012-2362-4

[11] Roff DA (1992) The evolution of life histories. New York: Chapman & Hall. 535 p.

[12] Stearns SC (1992) The evolution of life Histories. OxfordUK: Oxford University Press. 249 p.

[13] Bauernfeind SS, Fischer K (2007) Maternal body size as an evolutionary constraint on egg size in a butterfly. Evolution 61: 2374–2385. 17711470 10.1111/j.1558-5646.2007.00197.x

[14] Andersson M (1994) Sexual selection. Princeton: Princeton University Press. 599 p.

[15] Berven KA (1981) Mate choice in the wood frog, *Rana sylvatica* . Evolution 35: 707–722. 28563133 10.1111/j.1558-5646.1981.tb04931.x

[16] Verrell PA (1989) Male mate choice for fecund females in a plethodontid salamander. Anim Behav 38: 1086–1088.

[17] Gage JG (1998) Influence of sex, size, and symmetry on ejaculate expenditure in a moth. Behav Ecol 9: 592–597.

[18] Bergström J, Wiklund C, Kaitala A (2002) Natural variation in female mating frequency in a polyandrous butterfly: effects of size and age. Anim Behav 64: 49–54.

[19] Wherry T, Elwood RW (2009) Relocation, reproduction and remaining alive in an orb-web spider. J Zool 279: 57–63.

[20] Fitt GP (1990) Comparative fecundity, clutch size, ovariole number and egg size of *Dacus tryoni* and *D. jarvisi*, and their relationship to body size. Entomol Exp Appl 55: 11–21.

[21] Malmqvist B, Adler PH, Strasevicius D (2004) Testing hypotheses on egg number and size in black flies (Diptera: Simuliidae). J Vect Ecol 29: 248–256. 15707284

[22] Jones RE, Hart JR, Bull GD (1982) Temperature, size and egg production in a cabbage butterfly, *Pieris rapae* . Austral J Zool 30: 223–232.

[23] Berger A (1989) Egg weight, batch size and fecundity of the spotted stalk borer, *Chilo partellus* in relation to weight of females and time of oviposition. Entomol Exp Appl 50: 199–207.

[24] Rauter CM, Mcguire MJ, Gwartney MM, Space JE (2010) Effect of population density and female body size on number and size of offspring in a species with size-dependent contests over resources. Ethology 116: 120–128.

[25] Carriere Y, Roff DA (1995) The evolution of offspring size and number: a test of the Smith-Fretwell model in three species of crickets. Oecologia 102: 389–396. 28306850 10.1007/BF00329806

[26] Mousseau TA, Fox CW (1998) The adaptive significance of maternal effects. Trends Ecol Evol 13: 511–534. 10.1016/s0169-5347(98)01472-4 21238360

[27] Bonduriansky R, Day T (2009) Nongenetic inheritance and its evolutionary implications. Annu Rev Ecol Evol Syst 40: 103–125.

[28] Huck UW, Labov JB, Lisk RD (1987) Food-restricted first generation juvenile female hamsters (*Mesocricetus auratus*) affects sex ratio and growth of third generation offspring. Biol Reprod 37: 612–617. 3676408 10.1095/biolreprod37.3.612

[29] Naguib M, Gil D (2005) Transgenerational body size effects caused by early developmental stress in zebra finches. Biol Lett 1: 95–97. 17148137 10.1098/rsbl.2004.0277 PMC1629067

[30] Valtonen TM, Kangassalo K, Pölkki M, Rantala MJ (2012) Transgenerational effects of parental larval diet on offspring developmental time, adult body size and pathogen resistance in *Drosophila melanogaster* . PLoS ONE 7: e31611. 22359607 10.1371/journal.pone.0031611 PMC3281084

[31] Bonduriansky R, Head M (2007) Maternal and paternal condition effects on offspring phenotype in *Telostylinus angusticollis* (Diptera: Neriidae). J Evol Biol 20: 2379–2388. 17956399 10.1111/j.1420-9101.2007.01419.x

[32] Naguib M, Nemitz A, Gil D (2006) Maternal developmental stress reduces reproductive success of female offspring in zebra finches. Proc R Soc B - Biol Sci 273: 1901–1905. 10.1098/rspb.2006.3526 PMC1634771 16822750

[33] Macke E, Magalhães S, Khan HD-T, Luciano A, Frantz A, et al (2011) Sex allocation in haplodiploids is mediated by egg size: evidence in the spider mite *Tetranychus urticae* Koch. Proc R Soc B - Biol Sci 278: 1054–1063. 10.1098/rspb.2010.1706 PMC3049031 20926443

[34] McMurtry JA, Croft BA (1997) Life-styles of phytoseiid mites and their roles in biological control. Annu Rev Entomol 42: 291–321. 15012316 10.1146/annurev.ento.42.1.291

[35] Sabelis MW, Dicke M (1985) Long-range dispersal and searching behaviour. In: Helle W, Sabelis MW, editors. Spider mites. Their biology, natural enemies and control, vol 1B. Amsterdam: Elsevier. 141–160.

[36] Sabelis MW, Nagelkerke CJ, Breeuwer AJ (2002) Sex ratio control in arrhenotokous and pseudo-arrhenotokous mites. In: Hardy ICW, editor. Sex ratios. Concepts and research methods. Cambridge, UK: Cambridge University Press. 235–253.

[37] Sabelis MW, Nagelkerke CJ (1988) Evolution of pseudo-arrhenotoky. Exp Appl Acarol 4: 301–318.

[38] Amano H, Chant DA (1978) Some factors affecting reproduction and sex ratios in two species of predacious mites, *Phytoseiulus persimilis* Athias-Henriot and *Amblyseius andersoni* (Chant) (Acarina; Phytoseiidae). Can J Zool 56: 1593–1607.

[39] Walzer A, Paulus HF, Schausberger P (2006) Oviposition behavior of predatory mites: response to the presence of con- and heterospecific eggs. J Insect Behav 19: 305–320.

[40] Schausberger P, Hoffmann D (2008) Maternal manipulation of hatching asynchrony limits sibling cannibalism in the predatory mite *Phytoseiulus persimilis* . J Anim Ecol 77: 1109–1114. 18624737 10.1111/j.1365-2656.2008.01440.x

[41] Walzer A, Schausberger P (2011) Threat-sensitive anti-intraguild predation behaviour: maternal strategies to reduce offspring predation risk in mites. Anim Behav 81: 177–184. 21317973 10.1016/j.anbehav.2010.09.031 PMC3018599

[42] Amano H, Chant DA (1977) Life history and reproduction of two species of predacious mites, *Phytoseiulus persimilis* Athias-Henriot and *Amblyseius andersoni* (Chant) (Acarina; Phytoseiidae). Can J Zool 55: 1978–1983.

[43] Vanas V, Enigl M, Walzer A, Schausberger P (2006) The predatory mite *Phytoseiulus persimilis* adjusts patch-leaving to own and progeny prey needs. Exp Appl Acarol 39: 1–11. 16680562 10.1007/s10493-006-0024-0

[44] Stephens PA, Boyd IL, McNamara JM, Houston AI (2009) Capital and income breeding: their meaning, measurement and worth. Ecology 90: 2057–2067. 19739368 10.1890/08-1369.1

[45] Strodl MA, Schausberger P (2012) Social familiarity reduces reaction times and enhances survival of group-living predatory mites under the risk of predation. PLoS ONE 7 (8): e43590. 10.1371/journal.pone.0043590 PMC3425479 22927997

[46] Schausberger P (1997) Inter- and intraspecific predation on immatures by adult females in *Euseius finlandicus*, *Typhlodromus pyri* and *Kampimodromus aberrans* (Acari: Phytoseiidae). Exp Appl Acarol 21: 131–150.

[47] Krantz GW, Walter DE (2009) A Manual of Acarology. 3rd edition. LubbockUSA: Texas Tech University Press. 807 p.

[48] Croft BA, Luh HK, Schausberger P (1999) Larval size relative to larval feeding, cannibalism of larvae, egg or adult female size and larval-adult setal patterns among 13 phytoseiid mite species. Exp Appl Acarol 23: 599–610.

[49] Bühl A (2008) SPSS 16. Einführung in die moderne Datenanalyse. MünchenGER: Pearson Studium. 896 p.

[50] Schulten GGM (1985) Mating. In: Helle W, Sabelis MW, editors. Spider mites. Their biology, natural enemies and control, volume 1B. Amsterdam: Elsevier. 55–65.

[51] Crespi BJ (1989) Causes of assortative mating in arthropods. Anim Behav 38: 980–1000.

[52] Amano H, Chant DA (1978) Mating behaviour and reproductive mechanisms of two species of predacious mites, *Phytoseiulus persimilis* Athias-Henriot and *Amblyseius andersoni* (Chant) (Acarina; Phytoseiidae). Acarologia 20: 196–213.

[53] Dean JK (1981) The relationship between lifespan and reproduction in the grasshopper *Melanoplus* . Oecologia 48: 365–368. 10.1007/BF00346499 28309757

[54] Partridge L (1986) Sexual activity and lifespan, In: Collatz, K-G, Sohal RS, editors. Insect aging: Strategies and mechanisms. New York: Springer Verlag. 45–54.

[55] Delesalle VA (1986) Division of parental care and reproductive success in the zebra finch (*Taeniopygia guttata*). Behav Proc 12: 1–22. 10.1016/0376-6357(86)90066-5 24924533

[56] Zann RA (1996) The zebra finch: a synthesis of field and laboratory studies. New York: Oxford University Press. 352 p.

[57] Martins TLF (2004) Sex-specific growth rates in zebra finch nestlings: a possible mechanism for sex ratio-adjustment. Behav Ecol 15: 174–180.

[58] Walzer A, Schausberger P (2012) Body size matters in male lifetime reproductive success of the predatory mites *Phytoseiulus persimilis* and *Neoseiulus californicus* In: Schausberger P, Walzer A, Peneder S, editors. 7^th^ Symposium of the European Association of Acarologists: Acari in a changing world; program, abstracts, participants. p. 71.

[59] Fox CW, Czesak ME (2000) Evolutionary ecology of progeny size in arthropods. Annu Rev Entomol 45: 341–369. 10761581 10.1146/annurev.ento.45.1.341

[60] Vijendravarma RK, Narasimha S, Kawecki TJ (2010) Effects of parental larval diet on egg size and offspring traits in *Drosophila* . Biol Lett 6: 238–241. 19875510 10.1098/rsbl.2009.0754 PMC2865044

