# Correction: Intra- and Trans-Generational Costs of Reduced Female Body Size Caused by Food Limitation Early in Life in Mites

**DOI:** 10.1371/annotation/c8279f89-2414-4628-af2c-e5e030da11b5

**Published:** 2013-12-03

**Authors:** Andreas Walzer, Peter Schausberger

In the Funding Statement, the number of one of the grants from the Austrian Science Fund is incorrect. The Funding Statement should read: "This study was funded by the Austrian Science Fund (www.fwf.ac.at, P19824-B17 and P23504-B17). The funders had no role in study design, data collection and analysis, decision to publish, or preparation of the manuscript." 

